# Yield and Properties of Ethanol Biofuel Produced from Different Whole Cassava Flours

**DOI:** 10.5402/2013/916481

**Published:** 2013-01-21

**Authors:** F. T. Ademiluyi, H. D. Mepba

**Affiliations:** ^1^Department of Chemical/Petrochemical Engineering, Rivers State University of Science and Technology, 234 Port Harcourt, Nigeria; ^2^Department of Food Science and Technology, Rivers State University of Science and Technology, 234 Port Harcourt, Nigeria

## Abstract

The yield and properties of ethanol biofuel produced from five different whole cassava flours were investigated. Ethanol was produced from five different whole cassava flours. The effect of quantity of yeast on ethanol yield, effect of whole cassava flour to acid and mineralized media ratio on the yield of ethanol produced, and the physical properties of ethanol produced from different cassava were investigated. Physical properties such as distillation range, density, viscosity, and flash point of ethanol produced differ slightly for different cultivars, while the yield of ethanol and electrical conductivity of ethanol from the different cassava cultivars varies significantly. The variation in mineral composition of the different whole cassava flours could also lead to variation in the electrical conductivity of ethanol produced from the different cassava cultivars. The differences in ethanol yield are attributed to differences in starch content, protein content, and dry matter of cassava cultivars. High yield of ethanol from whole cassava flour is best produced from cultivars with high starch content, low protein content, and low fiber.

## 1. Introduction

Ethanol is used extensively as a solvent in the manufacture of varnishes and perfumes; as a preservative for biological specimens; in the preparation of essences and flavorings; in many medicines and drugs; as a disinfectant and in tinctures (e.g., tincture of iodine); as a fuel and gasoline additive (Columbia Encyclopedia). Ethanol has been produced from different sources in the past. The generally accepted sources of raw material for alcohol production from starch are cereal grains such as corn, wheat, rye, barley, milo (sorghum grains), rice, potatoes, apple wine, and others [1, 2]. 

The use of nonfood sources such as microalgae to produce ethanol gave low yield of ethanol when compared with food crops like sugar cane and cassava. The highest ethanol production using spirogyra algae fermented with *Z. mobilis *was 9.70% ethanol (v/v) with addition of *α*-amylase enzyme at 0.09 grams for 96 hours, while using spirogyra algae fermented with *S. cerevisiae *the highest ethanol production was 4.42% ethanol (v/v) with addition of *α*-amylase [3]. Therefore, Cassava starch is still a promising renewable resource in centuries to come as global reserves dwindles. In Nigeria, the national goal is to have a 10% blend that requires 1.27 billion litres of ethanol per year. Brazil, the world leading producer of this substitute, makes more than 120 million liters per year of ethanol from sugar cane and cassava. Promising ethanol returns, combined with higher crude oil prices throughout much of the year, have buoyed the demand for cassava in energy and alcohol production [4]. Also the carbohydrate content of cassava is higher than other root crop like potato, and so forth [5]. Ecofys 2007 reported that one tonne of fresh cassava roots yields 150 litres of ethanol and one tonne of dry cassava chips yields 333 litres of ethanol.

 Several authors have carried out production of ethanol from cassava via acid and enzyme hydrolysis [6–8] using only one cassava cultivar. Onitilo et al. [9] reported that there were significant differences in the starch content, amylase, amylopectin, and total titrable acidity of different cassava varieties which are more than forty types (40), hence there is a need to investigate the yield and properties of ethanol biofuel produced from different cassava cultivars. Ethanol handing guide [10] also revealed that the electrical conductivity, gum, and water cement, as well as particulate content, are important physical properties that must be checked in fuel ethanol before operation, the acidity, as well as hydrocarbon content, and flash point are also important properties that describe the quality of ethanol fuel before usage. 

Therefore, the objective of this work is to determine the yield and physical properties of biofuel (ethanol) produced from different whole cassava flours.

## 2. Materials and Methods

### 2.1. Materials and Equipments Used

Cassava cultivars: TMS 91/02324, TMS 92B/00061, TMS 92B/00068, TMS 98/0505, and TMS 98/0581, obtained from the International Institute of Tropical Agriculture (IITA) Onne., Rivers state Nigeria, refractometer, autoclave machine, conical flasks, spatula, industrial oven, pycnometer, thermostat, test tubes, thermometer, flash point apparatus, distillation apparatus, pH paper and meter, dry active yeast, yeast extract, potassium diphosphate, calcium chloride, magnesium sulphate, iron II sulphate (nutrients), sodium hydroxide for pH correction, sulphuric acid for hydrolysis, 95% pure ethanol (used to prepare standard curve for ethanol), deionized water, pipette, buffer solution, and U-tubes capillary viscometer.

### 2.2. Experimental Procedure

10 kg of each of the five cassava cultivars mentioned above were obtained from the International Institute of Tropical Agriculture (IITA), Onne. The roots were hand-peeled, washed separately, grated, and were pressed with a screw presser, the pressed roots were spread in separate trays in an open sun to remove initial moisture and dried in air oven to remove final moisture and, then, finally blended into fine particles as whole flour to increase the surface area.

#### 2.2.1. Preparation and Sterilization of Growth Media

A semisynthetic broth media was prepared using the following: potassium diphosphate, ammonium sulphate, calcium chloride magnesium sulphate, iron II sulphate, and yeast extract. All were dissolved in one liter of distilled water in a conical flask. The solution was placed into an autoclave at a temperature of 121°C for 15 minutes and at a pressure of 15 psia to destroy any impurity that might inhibit a microorganism in the system and allowed to cool to 40°C before it was brought out of autoclave. 

#### 2.2.2. Acid Hydrolysis of Starch

10 g of each of the whole cassava flour prepared from five different cassava cultivars (TMS 91/02324, TMS 92B/00061, TMS 92B/00068, TMS 98/0505, and TMS 98/0581) were added into 150 mL of 0.2 molar solution of diluted sulphuric acid in five conical flasks. The samples were allowed to stand for 90 mins, heated to 70°C, and were stirred continuously to allow uniform temperature by attaching a temperature controller to the thermocouple. The hydrolysate was allowed to cool to 30°C and neutralized to pH of 6-7 with a known amount 0.5 molar solution of sodium hydroxide. 

#### 2.2.3. Inoculation of Sample

In order to determine the optimum amount of mineralized media and yeast required to produce optimum amount of ethanol, varying amount of mineralized media (2.5 mL, 5 mL, and 7.5 mL) were added to on the hydrolysate of each of the five samples. The amount of active yeast required for fermentation was also varied. The mixture was turned into air tight containers (15 Nos, anaerobic system) after hydrolysis to enable fermentation. The effect of amount of cassava flour/acid ratio required to produce optimum yield of ethanol was also carried out by varying the cassava flour/volume of acid ratio. The yield of ethanol for the different whole cassava flours was monitored with fermentation period in hours for 6 days. 

#### 2.2.4. Distillation Process

The fermented mixture was heated using a thermostatic heater and distilled via simple double-binary distillation to obtain ethanol biofuel from the different cassava flours.

### 2.3. Determination of the Properties of Ethanol

Appearance: The distillate was observed visually, using visual test method.

#### 2.3.1. Ethanol Yield

Ethanol concentration was monitored using a refractometer via the refractive index method. The refractive index was determined with a refractometer (Bellingham and Stanley, England 1366A-3R). A calibration curve was obtained initially by diluting pure 98% ethanol in water to obtain different concentrations of ethanol and their corresponding refractive indexes obtained from the refractometer readings.

#### 2.3.2. Flash Point

This test was carried out using Pensky Martens flash point apparatus. The cup in the apparatus was dried. 50 ml of each sample (ethanol produced) was transferred into the flash point cup. The cup was fixed into the position in the apparatus assembled with thermometer, and the apparatus was switched on; the heat was controlled by a steady stirrer to maintain a uniform temperature while passing a small flame across the material every five seconds. The temperature at which the vapour first flashes with a blue flame was recorded as the flash point of the sample, after each test the cup was washed and dried before subsequent test. This was carried out twice for all the samples.

#### 2.3.3. pH Test

pH meter was first inserted in a buffer solution to standardize the apparatus then placed into the sample (ethanol) and the readings were obtained.

#### 2.3.4. Density and Specific Gravity Test

Empty pycnometer was weighed. The pycnometer was filled with sample (ethanol), the excess was wiped off, the weight was recorded, and the density calculated using the formula:

(1) $$\begin{array}{c} \text{Density}\;\;\left(\text{g}/\text{mL}\right)=\frac{\text{mass}}{\text{Volume}}. \end{array}$$

Secondly, distilled water was filled into the pycnometer, weighed and recorded. The specific gravity was calculated using the formula:

(2) $$\begin{array}{c} \text{Specific}\;\;\text{gravity}\;\;\left(\text{Spg}\right)=\frac{\text{density}\;\;\text{of}\;\;\text{Ethanol}}{\text{Density}\;\;\text{of}\;\;\text{Water}}. \end{array}$$

#### 2.3.5. Viscosity Test

50 ml of ethanol was turned into A-arm of U-tube capillary viscometer through the orifices to the marked point. A sucker was used to lift the sample to the B-arm of the capillary to the marked point. A stop watch was used to regulate the time it took the ethanol to return (flow) to the mark under the B-arm, and the time noted. Viscosity calibration curve was then used to convert viscosity in seconds to centistokes.

#### 2.3.6. Distillation Range Test

A known volume of the ethanol was turned into ASTM distillation flask, fixed at its position, A thermometer inserted at the top and the heater was switched on. A receiver (cylinder) was kept at the distillate recovery point. The temperature reading was taken at the first drop of the distillate. As the temperature increased, the distillate increased in volume until there was a decrease in distillate and at the last drop the final temperature and volume were noted.

#### 2.3.7. Electrical Conductivity Test

Hi 8033 digital electrical conductivity meter (Hanna Instrument, Portugal) was used. A known quantity of ethanol produced from the different cultivars was poured into a beaker. The electrode was immersed to the censor market point. The meter was turned on to the required calibrated units and the reading was taken for all the samples when a stable reading was established.

## 3. Results and Discussion

### 3.1. Effect of Quantity of Yeast on Ethanol Yield from Different Whole Cassava Flours

**Figure 1:**
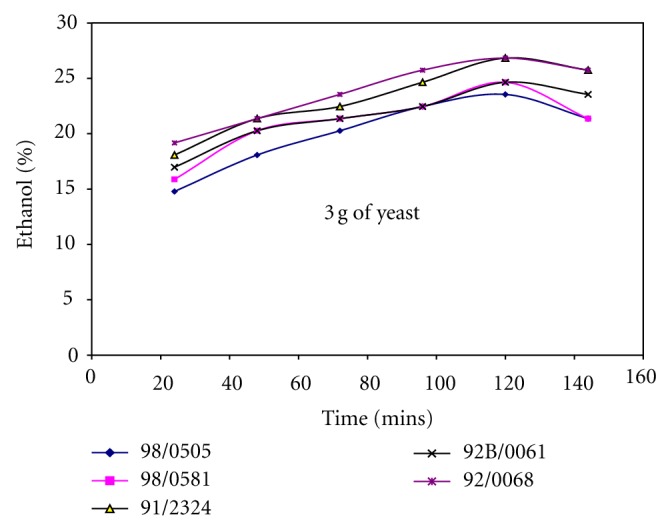
Ethanol yield for different cassava cultivars using 3 g of yeast.

**Figure 2:**
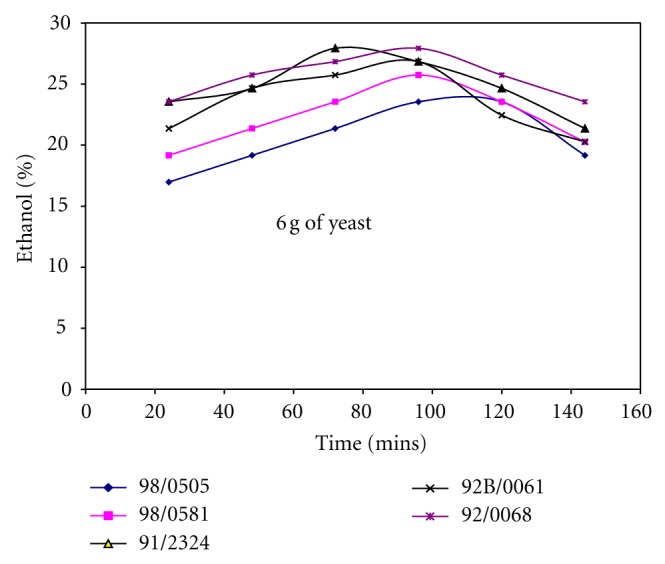
Ethanol yield for different cassava cultivars using 6 g of yeast.

**Figure 3:**
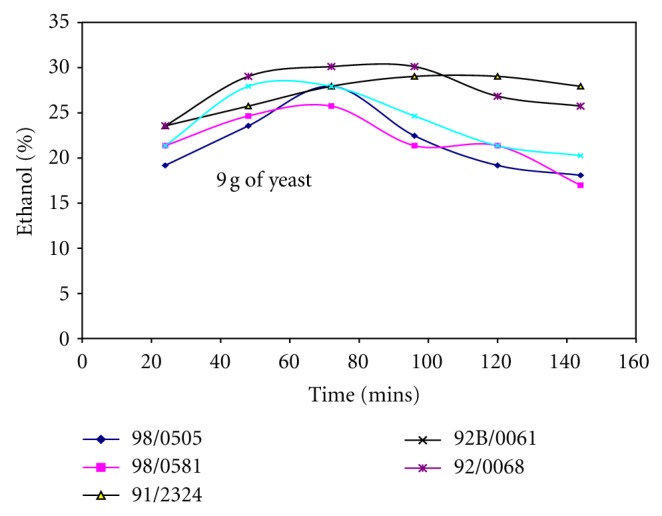
Ethanol yield for different cassava cultivars using 9 g of yeast.

Figures 1, 2, and 3 show the effect of the amount of yeast used on the yield of ethanol produced from different cassava cultivars. Generally, the yield of ethanol produced increased with fermentation time and later decreased. It shows that increasing the amount of yeast (3 g–9 g) along with the amount of mineralized media used also affects the yield of ethanol produced from different cassava cultivars. Comparing the fermentation times on Figures 1, 2, and 3; it was observed that increasing the quantity of yeast reduced the fermentation time, when 3 g of yeast was used the fermentation time was 120 hrs, while 6 g and 9 g of yeast required 72 hrs to produce over 25% ethanol. Although increasing the yeast to 9 g reduced the fermentation time and increased quantity of ethanol produced, it was observed the resultant solution produced after fermentation was too cloudy due to much yeast. Hence, 6 g of yeast to 5ml of mineralized media was preferred, this gave higher yield of ethanol at shorter fermentation time for all the cassava cultivars and was used as basis for production of ethanol from cassava in other studies carried out in this work.

Ethanol produced from whole cassava flours TMS 92/00068 and TMS 91/02324 shows consistent increase in ethanol yield irrespective of the quantity of yeast and mineralized media used as shown in Figures 1, 2, and 3. It was also observed that yield of ethanol produced from cassava flour TMS 98/0505 was the least as shown in Figures 1, 2, and 3 irrespective of the quantity of yeast and mineralized media used.

### 3.2. Effect of Whole Cassava Flour to Acid and Mineralized Media Ratio on the Yield of Ethanol Produced

It was observed from Figures 1, 2, and 3 that the yield of ethanol produced was between 15 and 30% using whole cassava flour to acid ratio of 1 : 15 (w/v) in the production of ethanol. Hence, quantity of whole cassava flour and acid used was optimized to obtain higher yield of ethanol.  Table 2 shows the effect of cassava flour/volume of media and acid ratio on the yield of ethanol produced for TMS 92B/00068. This analysis was carried out only for TMS 92B/00068 alone since this cultivar produced the highest yield of ethanol compared with other cultivars as shown in Figures 1, 2, and 3.  Table 2 shows that variation in the amount of cassava flour to volume of acid ratio as well as amount of cassava flour to volume of mineralized media ratio used has significant effect on ethanol yield. Increasing flour/volume of acid ratio increased the ethanol yield to an optimum value of 52% within 48-hour fermentation time and further increase in flour/acid and media ratio reduce the yield of ethanol produced. The optimum value of ethanol was produced using 0.333 g/mL (cassava flour/volume of acid) and 10 g/mL (ratio of cassava flour/volume of mineralized media) for TMS 92B/00068 fermented for 72 hrs.

### 3.3. The Properties of Ethanol Produced from Different Cassava

Table 3 shows the yield of ethanol produced from different whole cassava flours. The use of 0.333 g/mL of cassava flour/acid ratio of the different cassava cultivar produced 88.7–82.5% ethanol as shown in Table 3 after 72 hrs of fermentation and distillation. The volume of ethanol produced by different cassava cultivars varied significantly. TMS 92B/00068 had the highest ethanol produced (0.61 mL/g of cassava flour) after distillation, while TMS 98/0505 produced the least amount of ethanol (0.42 mL/g of cassava flour) as shown in Table 3. The density of ethanol produced from the different cassava cultivars was different after double distillation. Percentage ethanol by weight in water also differs significantly from the different cassava cultivars, with TMS 92B/00068 yielding up to 88.7% ethanol by weight in water after second distillation. Further distillation using fractional azeotropic distillation or dehydrating will be required to obtain ethanol of about 95–98% weight in water as shown in Table 1. All the ethanol produced was clear and colorless. The result in Table 3 shows that different cassava cultivars will not produce the same yield and density of ethanol.

Table 4 shows the distillation range of ethanol obtained from the different cassava cultivars. The distillation range of the ethanol produced from the different flours lies between 78−100°C with TMS 92B/00068 having the least value. This was expected since TMS 92B/00068 has the highest ethanol weight in water. Commercial (98%) ethanol has a boiling point of 78.4°C from Table 1. The difference in the distillation range of the different cassava cultivars was due to the variation in the densities of the ethanol produced from the different cassava cultivars. The flash point of different ethanol produced from the cassava whole flours were also presented in Table 4. The flash point value ranged between 15–24°C for ethanol weight in water of 88.65–82.49%. Standard ethanol from corn of 98% ethanol in water had a flash point of 13°C from Table 1, which can be obtained for ethanol from cassava using fractional distillation. TMS 92B/00068 still had the least flash point of 15°C compared with other cassava cultivars and will invariably have the highest heat of combustion. The lower the flash point, the better and faster the ignition of fuel. The difference in the flash point of the different cassava cultivars is also due to the variation in the densities of the ethanol produced from the different cassava cultivars also TMS 92B/00068 has more ethanol by weight in water. 

Table 5 shows the viscosity, electrical conductivity, and pH of ethanol from different whole cassava flours. The viscosity and pH varied slightly while the electrical conductivity of ethanol from the different cassava cultivars varied significantly. The electrical conductivity of fuel ethanol is an important parameter used when measuring fuel quality. A conductivity not more than 500 *μ*s/m is recommended [10]. The values obtained for cassava ethanol for all the cultivars is still within the required range of 500 *μ*s/m. TMS 92B/00068 and TMS 91/02324 have the highest electrical conductivity from Table 5 while TMS 98/0581 has the least, this result was expected since TMS 92B/00068 has high flash point compared with other cultivars. The work carried out by Adeniji et al. [13] on the mineral composition of five improved varieties of cassava showed that the mineral composition (sodium, magnesium, potassium, copper, zinc, iron, and phosphorus) of the five varieties of cassava studied varies significantly. This variation in mineral composition of the different whole cassava flours could also leads to variation in the electrical conductivity of ethanol produced from the different cassava cultivars.

Table 6 shows the average starch, dry matter, protein content and yield per hectare of fresh cassava cultivars used. TMS 92B/00068 and TMS 91/02324 had the highest average starch content and lowest % dry matter. From Tables 3 and 4, TMS 92B/00068 had the highest volume of ethanol produced after distillation and lowest flash point than other cultivars, while TMS 98/0505 and TMS 98/0581 had lower volume of ethanol produced after distillation. The high yield of ethanol produced using TMS 92B/00068 and TMS 91/02324 than other cultivars may be due to its high starch content. Cassava cultivar, TMS 91/02324, would be expected to produce more ethanol than TMS 92B/00068, because it had more starch content but from Table 6 the protein content of TMS 91/02324 is higher than the protein content of TMS 92B/00068, which shows that the low protein content also favours the yield of ethanol produced by the different cassava cultivars. In addition to high starch content, TMS 92B/00068 and TMS 91/02324 had low dry matter from Table 6. Low dry matter is a reflection of low fiber content. Low fiber content of cassava cultivars TMS 92B/00068 and TMS 91/02324 enhances the yeast to break down the hydrolysed starch to ethanol easily. The high cyanide content of TMS 92B/00068 and TMS 91/02324 did not affect the ethanol yield, cyanide content must have reduced during washing, dewatering, and drying of the fresh cassava roots to flour. 

## 4. Conclusion

The yield and properties of ethanol biofuel produced from different whole cassava flours was investigated. Yield and physical properties (distillation range, density viscosity, flash point, and electrical conductivity) of ethanol produced differ for different cassava cultivars. Optimum yield of ethanol was obtained using 0.333 g/mL (cassava flour/volume of acid) and 10 g/mL (ratio of cassava flour/volume of mineralized media) for TMS 92B/00068 fermented for 72 hrs. The differences in ethanol yield are attributed to differences in starch content, protein content, % dry matter, and mineral composition of cassava cultivars. Hence to produce high yield of ethanol with good physical and electrical properties from whole cassava flour, cassava cultivars with high starch content, low protein content, and low dry matter should be used, while the cassava cultivars with moderate starch content (<50%), high protein, and high fiber content can be used as food for human consumption and in food products.

## Figures and Tables

**Table 1:** Showing physical properties of commercial 98% ethanol.

Density and phase	0.789 g/cm³, liquid
Solubility in water	Fully miscible
Melting point	−114.3°C (158.8 K)
Boiling point	78.4°C (351.6 K)
Acidity (p*K*_a_)	15.9 (H^+^ from OH group)
Viscosity	1.200 mPa·s (cP) at 20.0°C

Source: Ethanol [11].

**Table 2:** Effect of variation of ratio of cassava flour/volume of mineralized media and acid on ethanol yield for TMS 92B/00068.

Experimental sets			Ethanol yield %	
	Ratio of cassava flour/volume of media (g/mL)	Ratio of cassava flour/volume of acid (g/mL)	24 hrs	48 hrs
a	4.0	0.1	15	18
	10.0	0.25	22	26
	20.0	0.30	42	46

b	2.0	0.0666	14	15
	5.0	0.167	22	24
	10.0	0.333	52	52
	15.0	0.5	28	30

c	2.0	1.33	12	9
	5.0	3.33	18	17
	10.0	6.67	32	38
	15.0	10.00	30	32

**Table 3:** Yield, volume, and density of ethanol produced from different cassava cultivars.

Name of cassava used	Density of first distillate (g/mL)	Density of second distillate (g/mL)	% Ethanol (weight in water)	Volume of ethanol (mL)/g of cassava flour produced after distillation	Appearance
TMS 92B/00068	0.9432	0.8206	88.7	0.60	Colorless
TMS 92B/00061	0.9506	0.8292	85.4	0.50	Colorless
TMS 91/02324	0.9409	0.8825	86.9	0.55	Colorless
TMS 98/0505	0.9572	0.8284	85.7	0.42	Colorless
TMS 98/0581	0.9463	0.8371	82.5	0.51	Colorless

**Table 4:** Distillation range and flash point of ethanol from different cassava cultivars.

Type of cassava used	Initial volume of distillate (mL)	Final volume of distillate (mL)	Distillation range (°C)	Flash point (°C)
TMS 92B/0068	82	72	78–99	15
TMS 91/02324	82	69	79–100	18
TMS 92B/0061	82	70	78–100	24
TMS 98/0505	82	67	79.5–100	19
TMS 98/0581	82	69	78–100	23

**Table 5:** Electrical conductivity, viscosity, and pH of ethanol from different whole cassava flours.

Type of cassava used	Electrical conductivity (*μ*s/m)	Viscosity (CST)	pH
TMS 92B/00068	329	1.99	6.11
TMS 91/02324	330	2.1	6.55
TMS 92B/0061	230	2.0	6.71
TMS 98/0505	300	2.02	6.68
TMS 98/0581	150	2.2	6.78

**Table 6:** Average starch, dry matter, protein content, cyanide, and yield per hectare of fresh cassava cultivars used.

Cassava cultivars	Starch content %	Protein %	Dry matter %	Cyanide level %	Yield of fresh cassava root tonnes/hectares
TMS 92B/0068	67.15	1.72	28.235	4.91	26.07
TMS 92B/0061	65.90	2.71	35.63	3.95	29.35
TMS 91/02324	67.75	4.55	27.615	6.81	21.09
TMS 98/0505	49.82	3.42	34.18	2.01	29.03
TMS 98/0581	47.04	3.22	35.22	2.60	25.67

Source: IITA Onne report [12].
